# Recombinant *Salmonella enterica* OmpX protein expression and its potential for serologically diagnosing *Salmonella* abortion in mares

**DOI:** 10.14202/vetworld.2023.1790-1795

**Published:** 2023-09-13

**Authors:** Sergey Borovikov, Anara Ryskeldina, Kanat Tursunov, Alfiya Syzdykova, Orken Akibekov

**Affiliations:** 1 Department of Microbiology and Biotechnology, Faculty of Veterinary and Animal Husbandry Technology, S. Seifullin Kazakh Agrotechnical Research University, 010000, Astana, Kazakhstan; 2 Department of Veterinary Medicine, Faculty of Veterinary and Animal Husbandry Technology, S. Seifullin Kazakh Agrotechnical Research University, 010000, Astana, Kazakhstan; 3 Laboratory of Immunochemistry and Immunobiotechnology, National Center for Biotechnology, 010000, Astana, Kazakhstan; 4 Research Platform of Agricultural Biotechnology, S. Seifullin Kazakh Agrotechnical Research University, 010011, Astana, Kazakhstan

**Keywords:** diagnostics, outer membrane proteins, recombinant antigens, *Salmonella* abortion in mares, *Salmonella enterica*, specific antibodies

## Abstract

**Background and Aim:**

*Salmonella* abortion in mares is caused by *Salmonella enterica* subspecies *enterica* serovar *abortus equi* infection and is characterized by premature (abortion) or non-viable fetus birth. Although all horses are susceptible to infection, the condition is more often clinically manifested in pregnant mares, with most abortions recorded in young females. In addition, nonspecific clinical disease signs and poorly sensitive and effective bacteriological diagnostic methods hinder rapid and reliable infection diagnoses. Immunochemical methods such as enzyme-linked immunosorbent assay (ELISA) and immunochromatography assays can facilitate effective and rapid diagnoses. However, they require highly specific and active antigens and antibodies. This study aimed to generate a recombinant *S. enterica* outer membrane protein X (OmpX) and evaluate its suitability for serological diagnosis of *Salmonella* abortion in mares**.**

**Materials and Methods:**

Outer membrane protein X from the *S. enterica* antigen was synthesized *de novo* and expressed in *Escherichia coli* using the pET28 vector. Transformed *E. coli* cells were cultured under different conditions to detect recombinant OmpX (rOmpX) expression, and rOmpX purification and refolding were both conducted using metal affinity chromatography. Refolded and purified rOmpX was characterized by western blotting, liquid chromatography with tandem mass spectrometry, and ELISA.

**Results:**

After optimized rOmpX expression, a 23 kDa molecular weight protein was identified. Amino acid sequence analysis using Mascot program suggested that these peptides were the OmpX protein from *S. enterica*. High specificity and diagnostic efficiency were recorded when rOmpX was used in ELISA against 89 serum samples from aborted and contact mares.

**Conclusion:**

Recombinant outer membrane protein, in comparison to the O antigen, demonstrated better diagnostic characteristics against sera from mares who aborted and contact horses.

## Introduction

*Salmonella* abortion in mares is an infectious disease of bacterial etiology and results in pregnancy termination and expulsion of dead or immature fetus from the uterus. In most mares, abortion occurs unexpectedly at 4–8 months. After abortion, increased body temperature and whitish mucus discharge from the vagina are observed. In a previous observational study (2–3 weeks), after abortion, animals were oppressed, refused to eat, and had a yellowish-brown vaginal discharge [1]. Based on clinical disease signs (abortion) and pathoanatomical changes in aborted fetuses, *Salmonell*a *enterica* subspecie*s enterica* serova*r abortus equi* infection was cited as a contributory agent [2]. However, a final diagnosis is often based on a bacteriological investigation of pathological material and specific pathogen detection [3].

The absence of specific clinical infections requires specific laboratory investigations. Furthermore, given often prolonged bacteriological analyses, several molecular methods have been developed, such as real-time polymerase chain reaction (PCR), and immunochemical methods such as enzyme-linked immunosorbent assay (ELISA) and immunochromatographic analysis, which are easy to perform and provide results within hours or even minutes [4–7]. However, immunogenic bacterial membrane proteins are required to ensure immunochemical test specificity. Several *S. enterica* surface antigens have been genetically and immunologically investigated, including flagellins, fliC and fljB, InvH and spvC, and the outer membrane protein X (OmpX) [8–11]. *Salmonella* OMPs are important cell wall components found on peptidoglycan layers in Gram-negative bacteria and are major regulators of small molecule membrane permeability. As the main component of outer bacterial membranes, *S. enterica* OmpX is immunodominant and an attractive target for serum antibody interactions [12].

This study aimed to generate a recombinant OmpX (rOmpX) antigen from *S. enterica* and evaluate its suitability for serologically diagnosing *Salmonella* abortion in mares using ELISA.

## Materials and Methods

### Ethical approval

All animal experiments were conducted after obtaining permission from the Local Ethical Committee of S. Seifullin Kazakh Agrotechnical Research University (No.1, August 26, 2020), in accordance with the Guidelines for the maintenance and care of animals (Interstate standard, GOST 34088-2017).

### Study period and location

The study was conducted from January 2022 to February 2023. Serum samples were collected from animals in private horse breeding farms and farmsteads in the Akmola, Karaganda, and Kostanay regions. The object of the study were mares that had previously experienced fetal abortion or had direct contact with them.

### Bacterial strains and plasmids

*Escherichia coli* DH5α and BL21 (DE3) cells (Novogene, Cambridge, UK), pGEM-TEasy plasmid (Promega, Madison, USA), and pET28 plasmid (Novogene) were used. *Escherichia coli* was grown in Luria–Bertani (LB) medium (Sigma-Aldrich, St. Louis, MO, USA).

### Recombinant protein plasmid construction, transformation, and expression

The OmpX reference amino acid sequence was obtained from the National Center for Biotechnology Information database (https://www.ncbi.nlm.nih.gov/protein/) (Accession No. NP_459810.1). Outer membrane protein X from *S. enterica* was codon optimized for *E. coli* using Vector NTI (https://vector-nti.software.informer.com/11.5/) and synthesized at the National Center for Biotechnology, Astana, Kazakhstan.

The synthesized DNA fragment was cloned into a pGEM-TEasy vector and transformed into chemo-competent *E. coli* DH5α cells. Positive colonies were selected using blue–white selection method and PCR screening of white colonies. Positive clones were selected and sequenced using an ABI PRISM® 310 Genetic Analyzer (Thermo Fisher Scientific, USA). The amino acid sequence encoding OmpX was cloned into the pET28 expression plasmid using *EcoRI* and *XhoI* restriction enzymes (Fermentas, Vilnius, Lithuania). The obtained expression vector was used to transform competent *E. coli* BL21 (DE3) cells, which were cultured on LB agar. After PCR screening, a single positive clone was inoculated into LB agar/kanamycin (50 μg/mL) and cultured. To determine optimal cultivation conditions positive colonies were cultivated in LB/kanamycin medium to an optical density (600 nm) of 0.5, and different isopropyl-β-D-thiogalactopyranoside (IPTG) (0.1–1 mM) concentrations were added. Cells were incubated at 25°C and 37°C, and sampling was performed every 2 h.

### Recombinant outer membrane protein purification and isolation

For protein isolation, cells were resuspended in 50 mM Tris–HCl (pH 7.4), 100 mM NaCl, and 0.1 mM ethylenediaminetetraacetic acid buffer supplemented with phenylmethylsulfonyl fluoride (0.1 mM), and disrupted using a UP200S ultrasonicator (Hielscher Ultrasonics GmbH, Teltow, Germany). Lysates were pelleted by centrifugation at 4000×g at 4°C for 10 min and resuspended in buffer plus 1 M urea, incubated further at 25°C for 30–40 min, and reprecipitated by centrifugation. The pellet was then resuspended in 8 M urea buffer and incubation and pelleting procedures were repeated. Recombinant outer membrane protein was isolated from the supernatant using 1 mL HisTrap HP columns (Cytiva, Uppsala, Sweden). A linear imidazole gradient (20–500 mM) was used to elute rOmpX and fractions were subjected to electrophoresis on 12% polyacrylamide gels, according to the Laemmli method [13]. Protein concentrations were determined using the Bradford assay. The rOmpX amino acid sequence was analyzed using liquid chromatography with tandem mass spectrometry (LC-MS/MS), as described previously by Borovikov *et al*. [14].

### Western blotting

After electrophoresis, proteins were transferred to nitrocellulose membranes using a Mini-PROTEAN Tetra cell system (Bio-Rad, Hercules, CA, USA). The transfer control was a pre-stained protein ladder (Thermo Fisher Scientific, Vilnius, Lithuania). Membranes were incubated in 1% bovine serum albumin (BSA) for 1 h at 25°C with constant stirring to block free zones. After washing 3 times in phosphate-buffered saline (PBS) containing Tween 20 (PBS-Tw), membranes were incubated at 4°C for 1 h with anti-6His-tag antibodies conjugated to horseradish peroxidase (HRP) (Qiagen, Hilden, Germany) (dilution 1:3000). After rewashing membranes, reactions were performed by adding 4-chloro-naphthol substrate (Sigma-Aldrich, St. Louis, MO, USA). Reactions were terminated by washing membranes in distilled water.

### Enzyme-linked immunosorbent assay

96-Well microplates (Nalge Nunk International, Rochester, NY, USA) were immobilized with 10 µg/mL *S. enterica* rOmpX or *Salmonella* lipopolysaccharide O antigen (PrioCHECK, Lelystad, Netherlands) in 0.1 M NaHCO_3_ (pH = 9). All incubation stages were performed at 37°C for 1 h. Plates were then washed 3 times in PBS-Tw. Free zones were blocked with 200 µL/well of 1% BSA. Serum samples from study animals were added at 1:100 dilution followed by a twofold dilution. An antihorse conjugated antibody labeled with horseradish peroxidase (Cusabio, Houston, TX, USA) 1:20,000) was added at 100 µL/well. Wells were then washed 3 times in PBS-Tw and then 3 times in PBS without Tween 20. Reactions were observed by adding 3,3′,5,5′-tetramethylbenzidine substrate (Bio-Rad, Hercules, CA, USA) at 100 μL/well. After 15 min, reactions were terminated by adding stop reagent and color development was recorded at 450 nm using a tablet spectrophotometer iMark (Bio-Rad, Tokyo, Japan).

ELISA sensitivity was determined on serum samples form aborted mares and confirmed using bacteriological and PCR methods in the laboratory of the National Veterinary Reference Center. For comparison, a commercial “*Salmonella* Latex Kit” (Liofilchem, Roseto d. Abruzzi, Italy) was used to detect specific antibodies. Specificity was determined in serum samples with the presence of antibodies against other pathogens (horse wash, infectious anemia of horses), which are found in the Republic of Kazakhstan.

### Statistical analysis

Enzyme-linked immunosorbent assay optical density values were analyzed and processed using GraphPad Prism 9.4.1 software 9.4.1. (https://www.graphpad.com/updates/prism-941-release-notes). Serum samples were analyzed in triplicate. Statistical data analyses were performed using t-tests (Student’s tests). The equality of the two populations was determined using F-tests. Data were represented by the mean ± standard error and significant differences were accepted at p < 0.05.

## Results

### Recombinant outer membrane protein construction, expression, and purification

**Figure-1:**
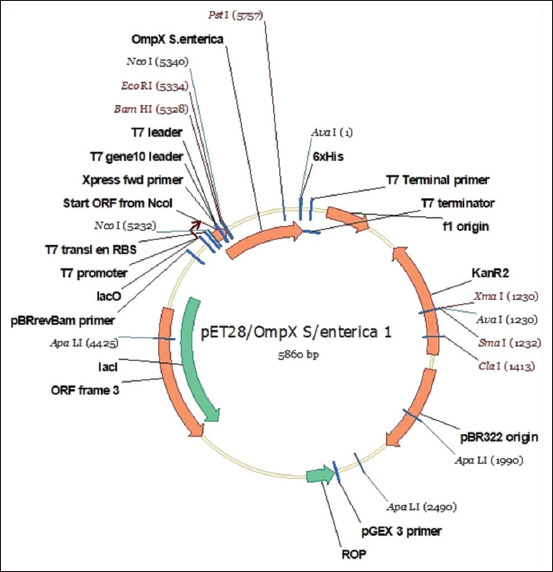
Schematic of the pET28/OmpX gene construct. rOmpX=Recombinant outer membrane protein.

Codon-optimized OmpX from the *S. enterica* antigen was cloned into pET28. The hexahistidine tag and restriction sites are shown (Figure-1).

*Escherichia coli* BL21 cells transformed with pET28/OmpX were cultured in LB/kanamycin medium containing different IPTG concentrations. Increased rOmpX expression was observed in samples every 2 h. After optimizing cultivation conditions, maximum protein expression occurred at 37°C, 0.1–0.2 mM IPTG, and a 6 h incubation period (Table-1). The recombinant protein was also localized to inclusion bodies (data not shown).

**Table-1:** Optimisation of rOmpX protein expression conditions.

IPTG concentration	0.1 mM (%)	0.2 mM (%)	0.5 mM (%)	1 mM (%)
pET28/OmpX				
25°°C	40	20	12	7
37°°C	78	76	53	32

rOmpX=Recombinant outer membrane protein, IPTG: Isopropyl-β-D-thiogalactopyranoside

**Figure-2:**
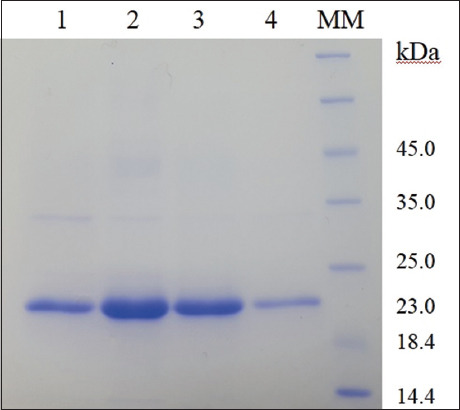
Sodium dodecyl sulfate–polyacrylamide gel electrophoresis of purified recombinant outer membrane protein. Lines 1–4: Purified recombinant protein fractions, MM=Molecular markers.

After HisTrap HP column chromatography, sodium dodecyl sulfate–polyacrylamide gel electrophoresis on eluted fractions indicated a protein of approximately 23 kDa molecular weight. The optimal imidazole elution concentration was 500 mM. The maximum protein concentration was ~950 µg/mL (Figure-2).

### Liquid chromatography with tandem mass spectrometry and western blot analysis of rOmpX

**Figure-3:**
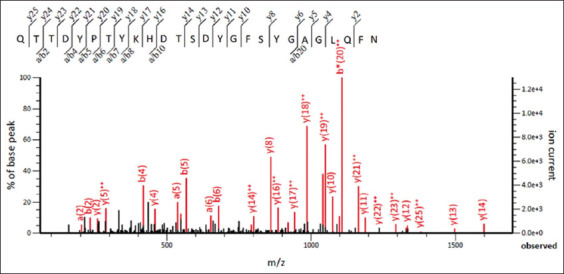
Tandem mass spectrometry spectrum of the fragmented peptides of recombinant outer membrane protein.

**Figure-4:**
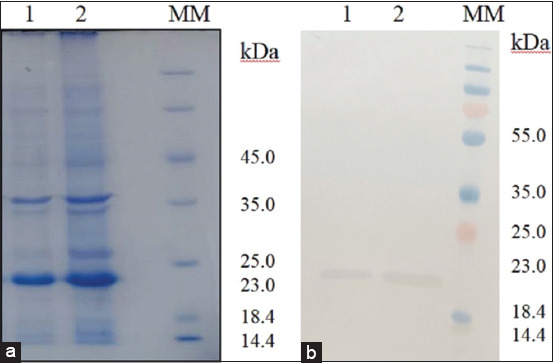
Polyacrylamide gel electrophoresis (a) and western blot (b) of recombinant outer membrane protein X with *Salmonella enterica*-positive serum. Lines 1–2: Recombinant outer membrane protein, MM=Molecular marker.

Recombinant outer membrane protein was examined using LC-MS/MS to determine its amino acid sequence; QTTDYPTYKHDTSDYGFSYGAGLQFN peptides were identified. Amino acid sequence analysis using Mascot suggested that these peptides were the OmpX protein from *S. enterica* (Figure-3). To ensure correct protein folding after affinity chromatography, urea concentrations were consistently reduced and specific rOmpX reactivity was determined by western blotting. Finally, the protein reacted with a positive sera pool from mares who aborted, indicating correct protein folding (Figure-4).

### Enzyme-linked immunosorbent assay analysis of pooled equine sera

**Figure-5:**
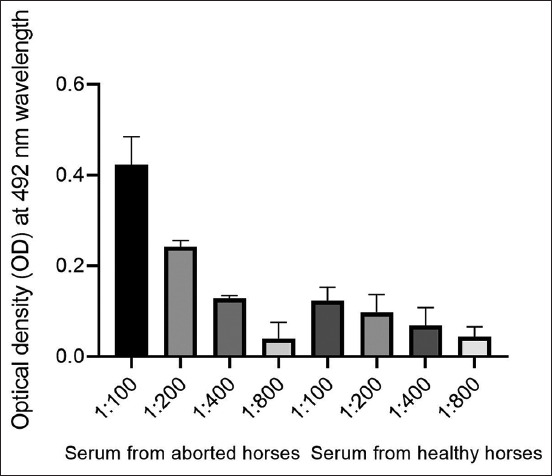
Titration histogram of six blood sera in enzyme-linked immunosorbent assay using *Salmonella enterica* recombinant outer membrane protein and standard deviations.

**Figure-6:**
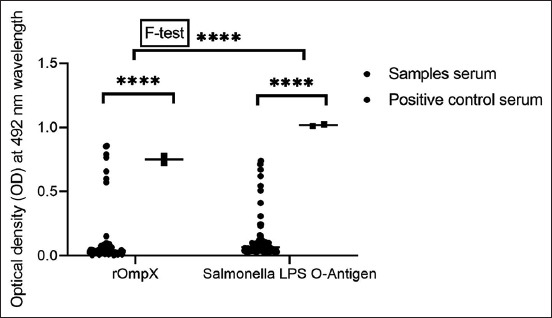
Optical density of the enzyme-linked immunosorbent assay reaction of the recombinant outer membrane protein and *Salmonella* O antigen with mare sera. Each symbol represents a separate serum sample.

Using rOmpX and a commercial *S. enterica* O antigen, 89 serum samples from mares who experienced abortions, and contact animals, were examined. For rOmpX-based ELISA, specific antibodies were established in eight samples and 12 samples for the O antigen. To determine standard deviation values, antibody titers were determined in six randomly selected serum samples from aborted and healthy mares at dilutions of 1:100–1:800. Enzyme-linked immunosorbent assay results showed antibodies against rOmpX at a titer of 1:800 (Figure-5). To establish average optical density values using different antigens, antibody titers were determined in all sera (Figure-6). Data analyses indicated the presence of antibodies to the OmpX antigen, and in some serum samples, antibody titers were higher when compared with the O antigen. Statistical analyses were performed using Student’s t-tests (*p = 0.0001) and F-tests (*p = 0.0001).

Enzyme-linked immunosorbent assay sensitivity when compared with the “*Salmonella* Latex Kit” was 114% for rOmpX and 171% for the O antigen. Specificity was confirmed by the absence of interaction with sera containing antibodies to antigens of heterologous infections in diagnostic titers.

## Discussion

*Salmonella enterica abortus equi* is responsible for *Salmonella* abortion in horses, which is characterized by premature birth (abortion) or non-viable fetus birth. After abortion, increased body temperature is associated with several complications, while metritis may also develop. Animals appear oppressed and refuse to feed, and complications such as arthritis, bursitis, and subcutaneous tissue abscesses may be manifested. To expedite rapid infection diagnoses, immunochemical methods are advised to identify specific antibodies in sera from sick animals. However, the diagnostic efficiency of these tests can considerably vary because the production of native antigens is difficult to standardize. [15].

Hence, rOmpX from *S. enterica* was generated to serologically diagnose *Salmonella* abortion in mares, which can also be used to obtain specific monoclonal antibodies. This *Salmonella* antigen diagnostic approach has been used in several studies [16, 17]. Outer membrane protein X from *S. enterica* was obtained using a *de novo* method, codon optimized for *E. coli* expression, cloned into an expression plasmid, and transformed into competent *E. coli* cells. Bacterial expression has been widely used to generate virus, bacterial, and eukaryotic diagnostic antigens [18, 19]. When we compared rOmpX amino acid sequence with the reference sequence, a complete match was established. Thus, a rOmpX protein (molecular weight = 23 kDa) was obtained. Correct protein refolding was confirmed by western blotting and ELISA with positive control sera.

Recombinant outer membrane protein activity and specificity were determined by indirect ELISA using 89 sera samples from mares that aborted and direct contact horses. The presence of specific antibodies was established in eight samples. Furthermore, specific antibodies were detected by ELISA based on a commercial O antigen in 12 samples. Despite the fact that fewer positive samples were detected using rOmpX, antibody titers in some cases were higher than the O antigen. Thus, rOmpX showed higher specificity when compared with the O antigen, which may have been due to increased lipopolysaccharide antigen cross-reactions. Our results agreed with other studies [20, 21] where recombinant antigens were successfully used to serologically diagnose salmonellosis in other animal and bird species.

## Conclusion

We successfully generated a *S. enterica* rOmpX protein. The protein actively interacted with specific antibodies in positive sera from infected animals at high titers. When rOmpX was used in ELISA against serum samples from mares who aborted, activity and specificity were established. However, before rOmpX is recommended for the serological diagnosis of *Salmonella* abortion in horses, further research is required. It will be important to compare rOmpX effectiveness against different *Salmonella* serotype antigens, which may improve diagnostic methods for this harmful infection.

## Authors’ Contributions

SB and KT: Designed and supervised the study and drafted and revised the manuscript. KT and AR: Preparation of genetic constructs, expression, and purification of recombinant protein. OA and AS: Sampling of sera from mares and working out the parameters of ELISA. SB: Data analysis. All authors have read, reviewed, and approved the final manuscript.
